# Structural Characterization and Antioxidant Activity of Chondroitin Sulfate Derived from *Channa argus* in Changbai Mountain

**DOI:** 10.3390/foods14213588

**Published:** 2025-10-22

**Authors:** Siyu An, Xu Zhang, Xiaoqin Wan, Wei Bing, Linlin Zhang, Yue Xiang, Wei An, Changhui Zhao

**Affiliations:** 1 Jilin Province Product Quality Supervision and Inspection Institute, Changchun 130103, China; 2 College of Food Science and Engineering, Jilin University, Changchun 130062, China

**Keywords:** *Channa argus*, structural characterization, chondroitin sulfate, antioxidant activity

## Abstract

Chondroitin sulfate (CS), commonly derived from animal cartilages, is a glycosaminoglycan with various bioactivities. This study employed an alkaline-enzyme method to prepare CS derived from *Channa argus* (referred to as CCS), using the heads and spines, which are food processing byproducts, as raw materials. Following this, we characterized its structure using HPLC, GPC, FTIR, and NMR and evaluated its antioxidant activity based on a TBHP-induced oxidative stress cell model. Structural analysis revealed that the CCS had a molecular weight of 5.09 kDa and was primarily composed of glucose and galactose units, featuring acetylated glucuronic acid–galactan chains with mixed α/β-glycosidic bonds. In vitro cellular antioxidant assays showed that CCS (80 μg/mL) significantly protected ARPE-19 cells against TBHP-induced oxidative damage by reducing ROS and MDA levels while enhancing the activity of typical antioxidant enzymes (CAT, SOD and GSH-Px). These findings indicate that CS derived from the byproduct of *Channa argus* processing has notable antioxidant properties and could serve as a promising food supplement for health applications.

## 1. Introduction

Chondroitin sulfate (CS) is a widely studied glycosaminoglycan (GAG) found extensively in the extracellular matrix of animal cartilaginous tissues and on cell surfaces [1]. It is composed of repeating disaccharide units of glucuronic acid (GlcA) and N-acetyl-galactosamine (GalNAc), which can be non-sulfated, mono-sulfated at the 4- or 6-positions of GalNAc, or disulfated at these positions and the 2-position of GlcA [2]. This structural diversity contributes to its multifunctional nature and broad nutraceutical and biomedical applications [3]. CS is known for its anti-inflammatory, anticoagulant, and anti-thrombotic properties [4], making it effective in treating osteoarthritis and cardiovascular diseases [5]. Additionally, CS promotes tissue repair and regeneration, particularly in cartilage and corneal healing. It also demonstrates potential in cancer therapy by inhibiting tumor cell proliferation and modulating the tumor microenvironment [6]. As a result, CS is widely incorporated into popular health products. With the aging population and the increase in chronic diseases, the market for CS products is expected to grow rapidly [7].

*Channa argus*, commonly known as the northern snakehead, belongs to the Perciformes order and is often referred to as black fish. This freshwater species, native to Asia and Africa, is highly valued for its large body size, few muscle bones, strong adaptability, and ease of cultivation. In China, it is particularly popular due to its perceived health benefits, such as wound healing and lactation promotion [8]. The species is rich in nutritional content and unique medicinal properties, making it a valued resource for health products.

CS is abundant in aquatic organisms and widely present in the bones, soft tissues, and cartilage of various aquatic organisms [9]. Traditional sources of CS have primarily been terrestrial mammals (such as pigs and cattle) and poultry (such as chickens), but these sources carry the risk of zoonotic diseases (such as bovine spongiform encephalopathy, foot and mouth disease) [10], which has led to concerns over the safety of CS derived from these animals [11]. Currently, shark cartilage is the main source of marine cartilage. Due to overfishing, global shark populations have declined by over 70% in the past 50 years [12], and the issue of shark conservation has attracted widespread international attention [13]. This situation underscores the importance of finding alternative sources of CS to meet the growing demand while ensuring sustainability.

Changbai Mountain, located in the southeast of Jilin Province, China, harbors a temperate primitive forest ecosystem and abundant species [14]. Notably, this region is distinguished by many natural animal resources such as the thriving population of *Channa argus* (northern snakehead). Despite the considerable research and application potential of *Channa argus*, studies focusing specifically on CS derived from this population remain relatively scarce. Therefore, our study aims to fill this gap by using head and spine bones of *Channa argus* (byproducts during the fish processing) as a raw material. We extracted CS derived from *Channa argus* (CCS) using an alkaline enzyme method and analyzed its structural characteristics. Additionally, we investigated its antioxidant activity to assess its potential as a valuable source of CS for nutraceutical and biomedical applications.

## 2. Materials and Methods

### 2.1. Materials and Reagents

The head and spine bones of *Channa argus* (byproducts during the fish processing) were from the Bai’shan aquaculture farms in Jilin Province. The ARP-19 cells were obtained from Shang’en Bio. The 2709 Alkaline Protease (CAS: 9014-01-1, 200 U/mg) was procured from Nanjing Duolai Biotechnology Co., Ltd. (Nanjing, China). The MTT (Methylthiazolyl Tetrazolium Chloride) was obtained from Beijing Solarbio Technology Co., Ltd. (Beijing, China). The DMEM medium and the Fetal Bovine Serum (FBS) were also purchased from the same company. The following kits were used in the study: Catalase (CAT) Assay Kit, Glutathione Peroxidase (GSH-PX) Assay Kit, Total Superoxide Dismutase (T-SOD) Assay Kit and Malondialdehyde (MDA) Assay Kit, all of which were obtained from Nanjing Jiancheng Bioengineering Institute (Nanjing, China). The Reactive Oxygen Species (ROS) Assay Kit was procured from Biyuntian Biotechnology Co., Ltd. (Shanghai, China).

### 2.2. Extraction of CS from Channa argus

The extraction method of CS was optimized based on the method reported by Wang et al. [15]. Specifically, the fresh fish heads and bones were steamed to remove excess muscle and other impurities. They were then soaked in ethanol overnight followed by drying in an oven at 65 °C (DGX-9073, Foma Experimental Equipment Co., Ltd., Shanghai, China). A total of 10 g of bone powder was mixed with 50 mM Na_2_CO_3_ solution at a ratio of 1:25 (*m*/*v*). The pH was adjusted to 7.0, followed by the addition of 200 μL of Savinase 6L. The mixture was stirred in a water bath at 55 °C for 4 h, and the enzyme was inactivated by heating in a boiling water bath at 100 °C for 10 min. After cooling to room temperature, alkaline protease 2709 was added to a final concentration of 5.4 mg/mL, and the reaction was carried out at 50 °C for 2 h, followed by inactivation at 100 °C for 10 min. The solution was cooled to 4 °C and centrifuged at 8000 r/min for 20 min. The supernatant was collected, and subsequently combined with one volume of 5% trichloroacetic acid, after which it was allowed to stand for 3 h. Following centrifugation, the supernatant was collected and subjected to precipitation with 3 volumes of ethanol at 4 °C overnight. The precipitate was collected by centrifugation, dissolved in 20 mM Na_2_SO_4_ solution, and then a 6% (*w*/*v*) cetylpyridinium chloride solution was added dropwise until no further precipitate formed. The precipitate was collected via centrifugation, dissolved in a 2 M NaCl solution in ethanol–water (100:15, *v*/*v*), and reprecipitated with 3 volumes of ethanol at 4 °C overnight. The resulting precipitate was collected by centrifugation, dissolved in distilled water, and dialyzed (8 kDa MWCO) for 24 h. The solution was subsequently concentrated and freeze-dried to obtain the final chondroitin sulfate product CCS.

### 2.3. CS Content Determination

Fifty microgram dried powdered CCS sample was weighed into a 50 mL centrifuge tube. Then, 10.0 mL of acetonitrile was added to disperse the sample evenly, followed by the addition of 10 mL of water. The mixture was shaken for 5 min and sonicated for 10 min to achieve complete dissolution. Subsequently, the solution was transferred into a 50 mL volumetric flask and diluted with water. After thorough mixing, the sample was returned to a 50 mL centrifuge tube and centrifuged at 4500 rpm for 5 min. The supernatant was filtered through a 0.22 μm organic filter membrane prior to analysis using a U3000 high-performance liquid chromatography (HPLC) system (Thermo Fisher Scientific, Bremen, Germany). The chromatographic conditions are as follows. Mobile phase: Acetonitrile + Sodium Pentane Sulfonate Solution (5 + 95). Chromatographic column: C18 (5 µm, 4.6 mm × 250 mm) chromatographic column. Flow rate: 0.6 mL/min. Detection wavelength: 192 nm. Injection volume: 10 μL.

### 2.4. Determination of CCS Molecular Weight

The molecular weight was determined based on a previous report [16] by the Ultrahydrogel 2000 (12 µm, 7.8 mm × 300 mm) series and Ultrahydrogel 500 (10 µm, 7.8 mm × 300 mm) using Alliance 2695 gel permeation chromatography (Waters, Milford, MA, USA).

### 2.5. Determination of Monosaccharide Composition

A pre-column derivatization procedure using (1-phenyl-3-methyl-5-pyrazolone, PMP) was carried out for analysis by high-performance liquid chromatography. Specifically, ten micrograms of the sample were mixed with 10 mL of 2 mol/L trifluoroacetic acid. The mixture was vortex-mixed, sealed, and hydrolyzed at 120 °C for 2 h in a high-temperature nitrogen-blanketed anaerobic oven. After hydrolysis, the sample was cooled to room temperature, dried under a stream of nitrogen, and reconstituted in 1 mL of distilled water. Then, 500 µL of 0.5 mol/L PMP methanol solution and 500 µL of 0.5 mol/L sodium hydroxide solution were added. The mixture was thoroughly mixed and incubated at 70 °C for 30 min for derivatization. Upon completion of the reaction, the mixture was neutralized with 500 µL of 0.5 mol/L hydrochloric acid. The solution was extracted three times with 3 mL of chloroform each time. The aqueous phase was collected and passed through a 0.45 µm filter membrane before analysis using a Thermo Scientific™ U3000 HPLC system (Thermo Fisher Scientific, Bremen, Germany). The chromatographic conditions are as follows. Column: ZORBAX Eclipse XDB-C18 separation column (4.6 × 250 nm, 5 μm). Mobile phase: phosphate-buffered solution (0.05 mol/L, pH 6.74)/acetonitrile (V:V = 83:17). Flow rate: 1 mL/min. Column temperature: 30 °C. Detection wavelength: 245 nm, UV detector. Injection volume: 10 μL.

### 2.6. Infrared Spectroscopy

Twenty micrograms of the dried sample and 180 mg of dried KBr were ground uniformly in an agate mortar. The mixture was loaded into a pellet die and manually pressed. The resulting pellet was then transferred carefully into the sample chamber. Fourier transform infrared spectroscopy was performed, and scanning was carried out within the wavenumber range of 4000–400 cm^−1^.

### 2.7. Nuclear Magnetic Spectroscopy

Twenty micrograms of the prepared CCS were dissolved in 0.5 mL of D_2_O, followed by freeze-drying. This process was repeated three times. The sample was then transferred into a nuclear magnetic resonance tube. ^1^H NMR and ^13^C NMR spectra were acquired at 25 °C using a Bruker 600 MHz NMR spectrometer.

### 2.8. Cell Culture

The human RPE cell line ARPE-19 (AC337713 ATCC) was cultured in DMEM medium supplemented with 10% fetal bovine serum and 1% penicillin–streptomycin. Cells were maintained at 37 °C in a humidified atmosphere containing 5% CO_2_.

### 2.9. Construction of Oxidative Damaged Retinal Pigment Epithelial Cells Induced by Tert-Butyl Hydroperoxide (TBHP)

To simulate oxidative stress-induced damage to retinal pigment epithelial cells, this study followed the methods of Deng et al. [17] and Gong et al. [18], treating ARPE-19 cells with TBHP and evaluating cell viability, ROS levels, and oxidative stress indicators using MTT assay, DCFH-DA fluorescent probe, and other methods. The details are as follows. ARPE-19 cell suspension (160 μL) was seeded into each well to achieve a density of 5 × 10^3^ cells per well, and the plate was cultured in a 37 °C incubator for 24 h. For the experimental groups, 40 μL of serum-free medium containing TBHP was added to each well to obtain final concentrations of 10, 20, 40, 80, 100, 150, 200, and 500 μmol/L, respectively. The control group received 40 μL of serum-free medium without TBHP, followed by incubation at 37 °C for 24 h.

### 2.10. MTT Assay

The cell suspension (160 μL) was added to each well of a 96-well plate and diluted to achieve a density of 5 × 10^3^ cells per well. The plate was incubated overnight at 37 °C with 5% CO_2_. Next, cells were treated with different compounds. Then, 50 μL of MTT reagent (5 mg/mL) was added to each well and incubated for 4 h. The supernatant was then carefully removed using a syringe, and 150 μL of DMSO was added to each well in the dark. The plate was continuously shaken for 15 min in a constant-temperature shaker. Finally, the 96-well plate was placed in a microplate reader, and the OD of the RPE cells was measured at 490 nm.

### 2.11. Determination of ROS Content

The release of ROS in RPE cells was measured using a 2,7-dichlorofluorescein diacetate (DCFH-DA) fluorescent probe. RPE cells in good logarithmic growth phase were inoculated into a 6-well plate and grouped for drug administration and culture following the same method described previously. After intervention, the cells were treated with DCFH-DA solution diluted in serum-free culture medium to a final concentration of 10 μmol/L and incubated at room temperature for 20 min. For the positive control, selected cells were pre-treated with Rosup (provided in the kit) for 30 min prior to probe loading. Fluorescence was observed and images were captured using the Leica DM4 B microscope. The light source was a mercury lamp, and the images were acquired using filter sets with an excitation wavelength of 488 nm and an emission wavelength of 525 nm.

### 2.12. Determination of Cellular Antioxidative Indicators

Cell culture and grouping methods were consistent with those described above. RPE cells in the logarithmic growth phase were inoculated into a 6-well plate before analysis. MDA and antioxidant enzyme activities (SOD, CAT and GPx) were measured under the nanostructure’s instructions by Nanjing Jiancheng bioengineering (Nanjing, China).

### 2.13. Statistical Analysis

All experimental data in this study are presented as the mean ± standard deviation (SD) of at least three independent experiments. Origin 2024 and SPSS 20.0 software were used to perform Duncan’s multiple test following analysis of variance (ANOVA). A value of *p* < 0.05 was considered statistically significant.

## 3. Results and Discussion

To ensure complete release of CS from bone meal and removal of protein impurities, we employed a dual enzymatic hydrolysis strategy (Savinase 6L and alkaline protease 2709) to ensure complete separation of CS from the protein core. After removing insoluble tissue residue by centrifugation, trichloroacetic acid was added to the supernatant to precipitate and remove any residual proteinase resistant acidic protein. Subsequently, CS was separated from soluble small molecule impurities such as salts, amino acids, and peptides using ethanol precipitation. In order to further purify CS and remove impurities such as neutral polysaccharides that may coprecipitate, we used a selective precipitation method with hexadecylpyridine chloride, which can form specific complexes with negatively charged GAGs (such as CS). Finally, the mixture was dissolved in a NaCl ethanol water solution and subjected to ethanol precipitation again to obtain high-quality CCS. The dual enzyme extraction method yielded a CCS of 15.96%.

A single symmetrical peak in the HPLC profile indicates a monodisperse molecular weight distribution with no other impurity peaks. To identify the type of CS in the sample, we conducted parallel analysis of commercially available CS standards under the same chromatographic conditions. The identification of the CS component in the sample is determined by directly comparing the retention time of its chromatographic peak with the retention time of the corresponding standard (Chondroitin sulfate C, derived from shark cartilage, CAS: 9007-28-7, EINECS number: 232-669-9). The standard curve with content (μg/mL) designated as the horizontal axis and peak area as the vertical axis is y = 13.39942x − 1.7890 (R^2^ = 0.99999), and the CS content that was determined is 81.3 g/100 g. The molecular weight of 5.09 kDa was determined by HPLC-GPC.

**Figure 1:**
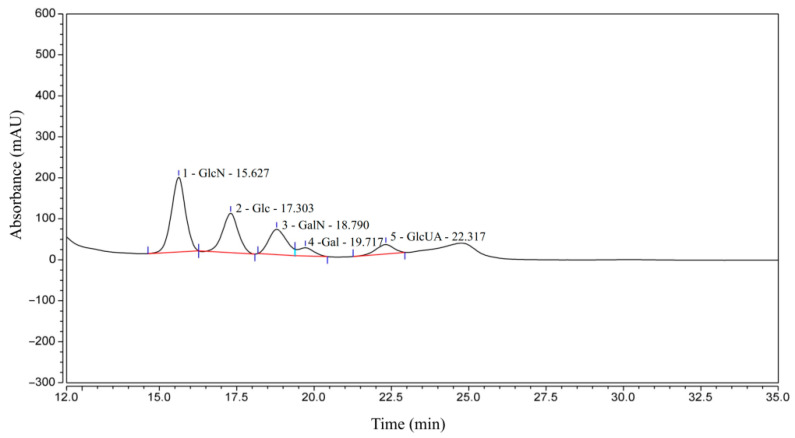
HPLC-UV results of CCS.

The sugar composition of CCS is shown in Figure 1 and Table 1. Specifically, GlcN is the most abundant component at 43.75%, followed by Glc at 25.51%, GalN at 18.41%, GlcUA at 6.86%, and Gal at 5.47%. The presence of monosaccharides, such as Glu and Gal, in the composition analysis can be primarily attributed to the following two factors. Firstly, the harsh acid hydrolysis conditions employed for monosaccharide determination readily cause the deacetylation and degradation of the core CS unit, Gal-NAc, generating Gal and GalN as major products, while GlcA can also be decarboxylated. Consequently, the substantial Gal detected is presumably derived primarily from the degradation of GalNAc, a well-documented complication in GAG analysis. Secondly, the potential presence of trace polysaccharide impurities is acknowledged, wherein minor GlcN might suggest the presence of trace hyaluronic acid or heparan sulfate, and trace glucose could stem from co-extracted glycogen. However, the overwhelming dominance of Gal (primarily derived from GalNAc) in the relative monosaccharide abundance strongly supports CS as the principal polysaccharide. Therefore, based on the comparison with the CS standard as well as its sugar composition, CCS used in this study contains CS, as well as small amounts of other GAGs, such as hyaluronic acid and heparan sulfate/heparin.

**Figure 2:**
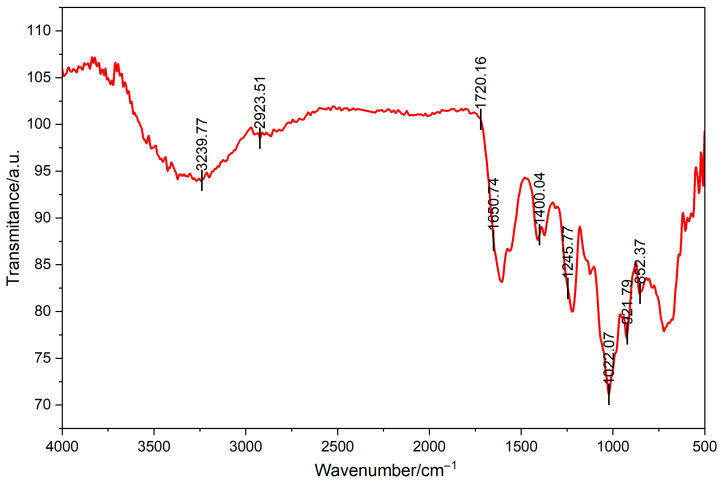
Fourier transform infrared spectrometry of CCS.

Data from Fourier transform infrared spectrometer were shown in Figure 2. A broad and strong absorption peak was observed in the range of 3400–3200 cm^−1^, attributed to the stretching vibration of hydroxyl groups (O-H) in polysaccharide molecules, indicating the presence of numerous hydroxyl groups in polysaccharides. The broadening of this peak may be attributable to intermolecular or intramolecular hydrogen bonding interactions. The absorption peak that appears in the range of 2930–2880 cm^−1^ is attributed to the stretching vibration of C-H, indicating the presence of alkyl structures in the polysaccharide. This peak is a characteristic of the C-H bond on the monosaccharide ring, indicating the presence of monosaccharides such as glucose and galactose structure within the sugar. The weak absorption peak that appears at 1740–1720 cm^−1^ may be attributed to the stretching vibration of carbonyl groups (C=O) in uronic acid or ester structures. The absorption peak that appears at a range of 1400–1300 cm^−1^ is attributed to the bending vibration of C-O-H, thereby further confirming the presence of hydroxyl groups in polysaccharides. The strong absorption peak that appears at wavenumbers between 1200 and 1000 cm^−1^ is attributed to the stretching vibration of C-O-C and C-O-H, indicating the presence of glycosidic bonds and hydroxyl groups in the polysaccharide. The absorption peaks that are observed at 920 cm^−1^ and 850 cm^−1^ may be attributed to the characteristic vibrations of α-glycosidic bonds and β-glycosidic bonds, respectively. The spectrum shows characteristic absorption peaks of CS. Notably, the strong absorption peak at ~1245 cm^−1^ attributed to the S=O stretching vibration of the sulfate ester, and the peak at ~850 cm^−1^ corresponding to the C-O-S stretching vibration characteristic of C4-sulfation in galactosamine residues. As demonstrated in the preceding analysis, the polysaccharide is predominantly composed of glucose and galactose moieties, and the glycosidic bond type may be a combination of α- and β-type.

**Figure 3:**
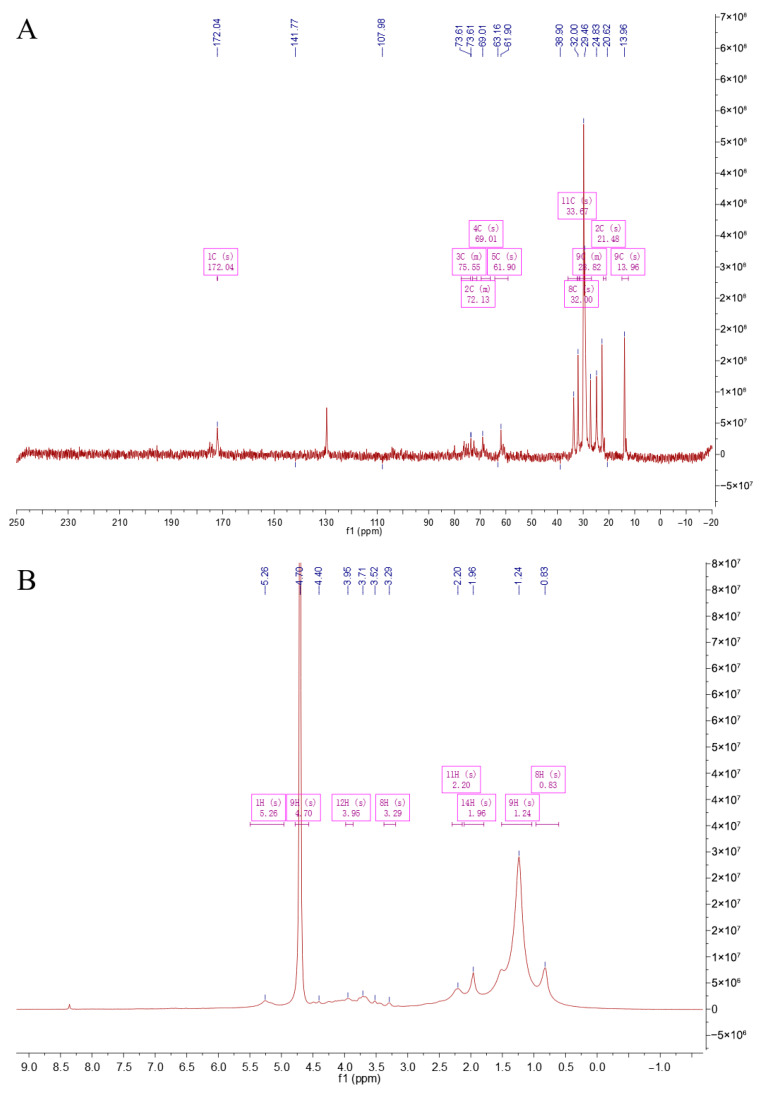
NMR spectroscopy of CCS. (**A**) C-spectrum; (**B**) H-spectrum.

NMR analysis is an important means of studying the structure of polysaccharides, which was used to detect the monosaccharide composition, glycosidic bond types, connection sequence, and spatial configuration [19]. Figure 3A is a ^13^C NMR spectrum. The singlet (1C, s) at a chemical shift of 172.04 ppm is a characteristic peak of carboxyl carbon, corresponding to the carboxyl carbon of uronic acid in CS. The peaks at 141.77 ppm and 107.98 ppm may correspond to carbons on the sugar ring affected by special electronic effects. The peaks in the regions such as 75.55 ppm (3C, m), 72.13 ppm (2C, m), 69.01 ppm (4C, s), and 61.90 ppm (5C, s) correspond to carbons connected with oxygen on the sugar ring. The peaks at relatively higher fields such as 33.97 ppm (11C, s), 32.00 ppm (8C, s), 29.82 ppm (9C, m), 21.45 ppm (2C, s), and 13.96 ppm (9C, s) correspond to alkyl carbons. Figure 3B is a ^1^H NMR spectrum. The singlet (1H, s) at 5.26 ppm corresponds to the methine hydrogen connected with oxygen on the sugar ring. The singlet (9H, s) at 4.70 ppm involves the hydrogens on the glucosamine ring. The peaks such as 3.95 ppm (12H, s) and 3.29 ppm (8H, s) correspond to other methylene or methine hydrogens on the sugar ring. The peaks at relatively higher fields such as 2.20 ppm (11H, s), 1.96 ppm (14H, s), 1.24 ppm (9H, s), and 0.83 ppm (8H, s) correspond to the hydrogens in the alkyl part. Overall, from the peak positions, integrations, and splitting patterns of ^1^H NMR and ^13^C NMR, there are characteristic carbon–hydrogen signals related to uronic acid, glucosamine, and sulfation in the structure of CS, indicating that the sample CCS contains the characteristic structural units of CS.

**Figure 4:**
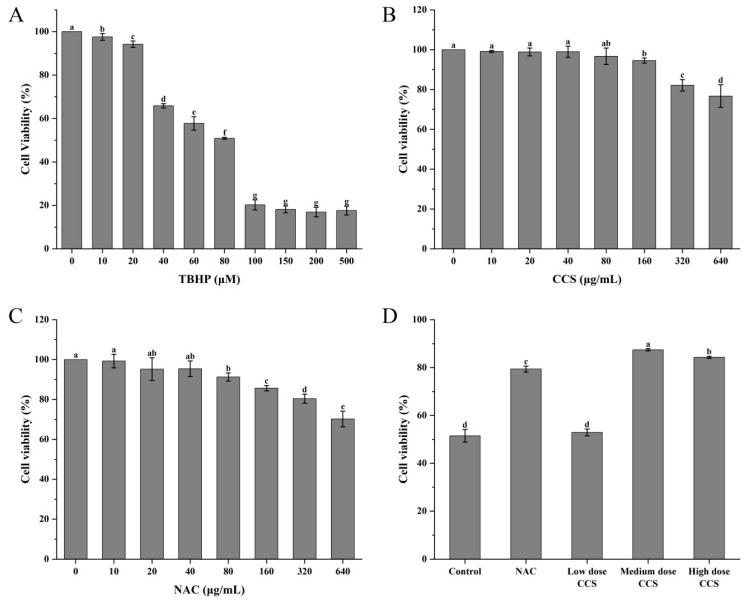
The effect of different treatments on the viability of ARPE-19 cells. (**A**) TBHP; (**B**) CCS; (**C**) NAC; (**D**) different doses of CCS. Low dose: 40 μg/mL; medium dose: 80 μg/mL; and high dose: 160 CCS. Different letters indicate significant differences (*p* < 0.05).

Within the concentration range of 0 to 500 μmol/L, TBHP significantly reduced cell survival rate with increasing concentration. It has been demonstrated that medium-dose CCS (80 μg/mL) exerts a significant inhibitory effect on TBHP-induced oxidative damage to RPE cells. In addition, its safety profile is superior to that of the classical antioxidant NAC. At a concentration of 80 μM, the cell survival rate was found to be approximately 50%, suggesting that TBHP at this concentration can induce oxidative damage to cells with a high degree of effectiveness. Consequently, it is deemed appropriate for subsequent experiments evaluating antioxidant activity (Figure 4A). When the concentration of CCS was between 0 and 500 μg/mL, CCS at 0 to 80 μg/mL had almost no effect on cell survival rate, with a cell survival rate exceeding 90%. However, an increase in the concentration of CCS to 320 μ g/mL and 640 μ g/mL resulted in a decline in cell survival rate, indicating a cytotoxic effect (Figure 4B). It has been demonstrated that N-acetylcysteine (NAC) exerts a concentration-dependent negative effect on cell viability. The presence of NAC, at concentrations ranging from 0 to 40 μg/mL, exerts minimal influence on cell viability. However, elevated concentrations of NAC have been observed to elicit a reduction in cell viability, thereby demonstrating cytotoxic effects (Figure 4C). A comparative analysis reveals that, in contrast to NAC, CCS exerts a comparatively negligible influence on cell viability. In comparison with the control group, low-dose CCS did not demonstrate a significant improvement in cell survival rate. However, the medium-dose CCS group exhibited a significant increase in cell survival rate, indicating its effective antioxidant protection effect. Despite the elevated cell survival rate observed in the high-dose CCS group, it remained lower than that of the medium-dose group, suggesting the potential for cellular toxicity (Figure 4D). It is evident that, in general, the present findings suggest that CCS exerts a protective effect on oxidative damage to cells, at moderate doses at least. However, at low doses, the protective effect is weaker, and at high doses, it is reduced due to toxicity.

**Figure 5:**
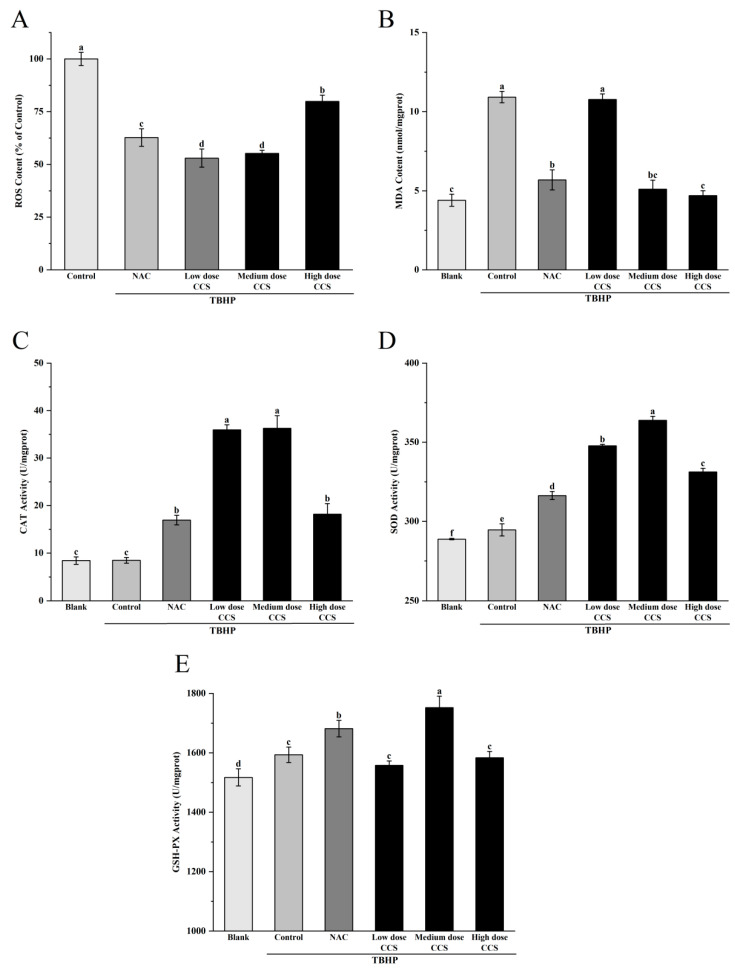
The effect of different treatments on the antioxidant activity of ARPE-19 cells induced by TBHP-induced oxidative damage. ROS content: (**A**), MDA content: (**B**), CAT Activity: (**C**), SOD Activity: (**D**), GSH-PX Activity: (**E**). Different letters indicate significant differences (*p* < 0.05).

Figure 5A shows the effect of different treatments on ROS content based on the ROS content of the control group (100%). The results showed that compared with the control group, the ROS content in both the NAC treatment group and the CCS treatment group decreased. The CCS group showed a dose-dependent decay. With the increase in CCS concentration, the ROS content increased, with higher ROS content in the high-dose CCS group and little difference in ROS content between the low and medium doses. This indicates that NAC and CCS have significant antioxidant activity and can effectively reduce ROS content, which is similar to the results of Huang et al. [20]. Medium-dose CCS (80 μg/mL) can effectively resist TBHP-induced oxidative damage of RPE cells, and its safety is better than that of classical antioxidant NAC. Compared with NAC, CCS has a relatively greater impact on ROS, indicating that CCS has stronger antioxidant capacity than NAC. Figure 5B shows that the blank group had the lowest MDA content, indicating a lower degree of cellular lipid peroxidation under normal physiological conditions. The MDA content in the control group significantly increased, indicating a significant increase in cellular lipid peroxidation after TBHP induction. The MDA content in both the NAC group and CCS group was lower than that in the control group, indicating that both have antioxidant effects. The MDA content in the low-dose NAC, CCS group was lower than that in the control group, indicating a certain antioxidant effect, but the effect was weaker. The MDA content rapidly decreased and the antioxidant effect was significant in the medium-dose CCS group. The MDA content in the high-dose CCS group continued to decrease, but the magnitude was small, slightly lower than that in the medium-dose group, and similar to the MDA content in the blank group, almost completely eliminating lipid peroxidation. The overall results indicate that CCS has antioxidant effects, similar to the results of Bae Jin Ha et al. [21], with the high-dose group showing the best effect and significantly reducing MDA content. Figure 5C shows that the blank group had the lowest CAT activity, indicating a lower baseline level of cellular CAT activity under normal physiological conditions. The CAT activity in the control group remained almost unchanged, indicating that after TBHP induction, intracellular oxidative stress was lower. The increase in CAT activity in the NAC group indicates that NAC, as an antioxidant, can enhance intracellular CAT activity. The CAT activity in the low-dose CCS group was significantly higher than that in the control group, indicating that low-dose SHI can activate CAT activity. The moderate-dose CCS group had the highest CAT activity, indicating that moderate dose SHI has a significant activating effect on CAT activity; The high-dose CCS group showed a decrease in CAT activity, but still higher than the control group and NAC group, indicating that the CCS high-concentration group still has a certain activating effect and stronger antioxidant capacity than the NAC group. Overall, CCS can activate CAT activity, similar to the results of Du et al. [22], with the medium-dose group showing the best effect. Figure 5D shows that the blank group had the lowest SOD activity, indicating a lower basal level of cellular SOD activity under normal physiological conditions. The SOD activity in the control group increased, indicating that TBHP-induced intracellular oxidative stress was enhanced, and SOD activity was activated to cope with oxidative damage. The further increase in SOD activity in the NAC group indicates that NAC, as an antioxidant, can enhance intracellular SOD activity. The SOD activity in the low-dose CCS group was significantly higher than that in the control group, indicating that low-dose CCS can activate SOD activity. The SOD activity was highest in the medium-dose CCS group, indicating that the medium-dose CCS has a significant activating effect on SOD activity. The SOD activity in the high-dose CCS group decreased, but remained higher than that in the control group and NAC group, indicating that the high concentration CCS group still had a certain activating effect, but the effect was weaker than that in the medium-dose group. Overall, CCS can activate SOD activity, similar to the results of Ajisaka et al. [23], with the medium-dose group showing the best effect. Figure 5E shows that the blank group had the lowest GSH-Px activity, indicating a lower basal level of antioxidant enzyme activity in cells under normal physiological conditions. The control group showed an increase in GSH-Px activity, indicating that after TBHP induction, intracellular oxidative stress was enhanced and GSH-Px activity was activated to cope with oxidative damage. The further increase in GSH-Px activity in the NAC group indicates that NAC, as an antioxidant, can enhance intracellular GSH-Px activity. The GSH-Px activity of the low-dose CCS group was similar to that of the control group, indicating that the effect of low-dose CCS on activating GSH-Px activity was weaker. The highest GSH-Px activity was observed in the medium-dose CCS group, indicating that the medium-dose CCS has a significant activating effect on GSH-Px activity. The high-dose CCS group showed a decrease in GSH-Px activity, similar to the control group, indicating that the high-concentration CCS group still has a certain activating effect, but the effect is weaker than that of the medium-dose group. Overall, CCS can activate GSH-Px activity, similar to the results of Yin Wang et al. [24], with the medium-dose group showing the best effect. Overall, CCS has a protective effect on cell membranes within a certain concentration range, with the low- and high-dose groups showing the best results.

**Figure 6:**
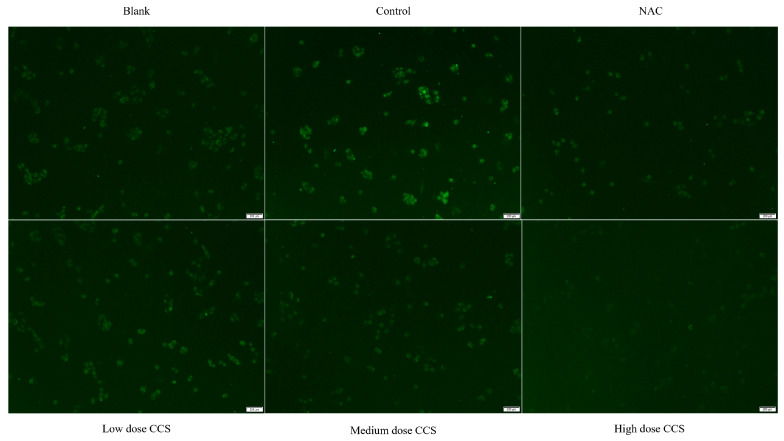
Fluorescence microscope photography results.

The fluorescence microscope photos provide a more intuitive representation of our experimental results (Figure 6). The blank group showed almost no fluorescence and low ROS levels, while the control group had high fluorescence intensity, indicating high ROS levels. The fluorescence intensity of both NAC and CCS groups significantly decreased, indicating a decrease in ROS levels. In the NAC group, the fluorescence intensity of low- and medium-dose CCS was similar to that of the blank group, with lower ROS levels. The fluorescence intensity of the high-dose CCS group was the lowest, lower than that of the blank group, indicating that high-dose CCS has an inhibitory effect on reactive oxygen species. Overall, high-dose CCS showed the best effect in reducing intracellular ROS levels, demonstrating strong antioxidant activity.

Based on a comprehensive analysis of antioxidant activity, CCS demonstrates significant dose-dependent antioxidant effects against TBHP-induced cellular damage. At 80 μg/mL, CCS optimally preserves cell viability (>90%) and exhibits potent ROS-scavenging activity, reducing ROS levels comparably to the positive control NAC. This dose also maximally activates antioxidant enzymes (CAT, SOD, GSH-Px) and suppresses lipid peroxidation (MDA), confirming enhanced cellular defense mechanisms. However, high-dose CCS (>320 μg/mL) induces cytotoxicity, diminishing cell survival and partially attenuating antioxidant enzyme activation despite further lowering ROS and MDA. NAC similarly reduces oxidative markers but exerts stronger negative effects on cell viability at higher concentrations. Fluorescence microscopy visually corroborates ROS suppression, with high-dose CCS showing the strongest inhibition. Thus, CCS exhibits strong antioxidant activity. Its antioxidant efficacy peaks at medium doses, balancing ROS clearance, enzyme induction, and cytoprotection without toxicity.

## 4. Conclusions

CCS is an acetylated glucuronic acid galactan, whose main chain is connected by a mixture of glucose and galactose through α/β-glycosidic bonds, containing glucuronic acid components and a significant hydrogen bonding network. It does not primarily contain conventional GlcA and GalNAc [25]. Based on our highly specific extraction and purification protocol for sulfated glycosaminoglycans, along with analytical data (monosaccharide composition dominated by Gal and GlcA, and FT-IR spectrum consistent with CS standards as well as NRM results), we have strong justification to attribute the observed biological activity predominantly to CS. Related studies have found that the core mechanism by which CS and its low-molecular-weight derivatives upregulate antioxidant enzymes is achieved through the activation of the Nrf2/Keap1 signaling pathway. Under oxidative stress conditions, CS modifies the redox-sensitive sites of Keap1 protein (such as cysteine residues) to promote the dissociation and translocation of Nrf2 from Keap1 to the nucleus, which then binds to antioxidant response elements and initiates the transcriptional expression of downstream antioxidant enzyme genes, including CAT, SOD, GSH-PX, and heme oxygenase-1 (HO-1) [26].

Conventional CS is typically composed of repeating disaccharide units of GlcA and GalNAc, with sulfation at the 4-O or 6-O positions of GalNAc [27]. Our analysis demonstrated that CCS exhibits CS characteristics with a mixture of α/β-glycosidic bonds. The presence of acetyl groups and the mixed glycosidic bond configuration may enhance its free radical scavenging capacity and improve its ability to modulate cellular antioxidant defense systems [28], as evidenced by its potent ROS-scavenging and enzyme-activating effects observed in our study. Moreover, marine-derived CS, such as that from shark cartilage, often shows higher molecular weights and more complex sulfation patterns [29]. However, CCS has a relatively low molecular weight, which may facilitate better cellular uptake and bioactivity [30]. These structural and functional distinctions highlight the potential of CCS as a sustainable and effective alternative to CS from endangered or disease-prone animal sources.

This study successfully isolated CCS in Changbai Mountain for the first time. And we comprehensively used HPLC, GPC, FTIR, and NMR techniques to confirm the unique structural characteristics and discovered its potent antioxidant activity. Compared with other related studies [31], the innovation of this study lies in the improvement of extraction methods, detailed characterization of structural features, and significant antioxidant activity. The alkaline enzyme method ensured the structural integrity of CCS, providing a foundation for subsequent biological activity research. This not only provides an environmentally friendly solution to replace terrestrial mammals and endangered sharks, but also addresses the risk of zoonotic diseases and resource conservation issues. It also provides a new source for health products made from CS, which is beneficial for alleviating population aging and chronic diseases. The industrial potential of CCS is promising. Its by-products (bones, skin, viscera) are rich in high-value components like proteins, collagen, and CS, which are often discarded, causing waste and environmental issues [32]. With proper processing, these can be converted into valuable food and pharmaceutical ingredients, reducing waste and bringing economic benefits to the fish processing industry. Thus, *Channa argus* by-products hold significant potential for industrial development and merit further research [33].

Subsequent research endeavors may entail the optimization of alkaline-enzyme extraction of CCS from fish by-products with the objective of enhancing yield while preserving its structural integrity. Further studies could clarify how CCS’s unique α/β-glycosidic bonds and acetyl groups regulate the molecular pathway via targeted cell models and mouse models. The development of pilot-scale processing for CCS will facilitate its industrial application and promote the valorization of aquatic waste.

## Figures and Tables

**Table 1:** HPLC-UV quantitative analysis results of CCS.

Serial Number	Peak Name	Retention (min)	Peak Area (mAU·min)	Peak Height (mAU)	Peak Area Ratio (%)	Peak Height Ratio (%)
1	GlcN	15.627	96.837	182.150	43.75	47.62
2	Glc	17.303	56.455	96.227	25.51	25.15
3	GalN	18.790	40.752	61.495	18.41	16.08
4	Gal	19.717	12.113	19.926	5.47	5.21
5	GlcUA	22.317	15.177	22.745	6.86	5.95
Sum			221.334	382.544	100.00	100.00

Notes: GlcN = Glucosamine; Glc = Glucose; GalN = Galactosamine; Gal = Galactose; GlcUA = Glucuronic acid.

## Data Availability

The original contributions presented in this study are included in the article. Further inquiries can be directed to the corresponding authors.
